# Potentiometric Electronic Tongue for Quantitative Ion Analysis in Natural Mineral Waters

**DOI:** 10.3390/s22166204

**Published:** 2022-08-18

**Authors:** María Cuartero, Alberto Ruiz, Manuel Galián, Joaquín A. Ortuño

**Affiliations:** 1 Department of Chemistry, School of Engineering Science in Chemistry, Biochemistry and Health, KTH Royal Institute of Technology, Teknikringen 30, SE-100 44 Stockholm, Sweden; 2 Department of Informatics and Systems, University of Murcia, 30100 Murcia, Spain; 3 Department of Analytical Chemistry, University of Murcia, 30100 Murcia, Spain

**Keywords:** water sample characterization, potentiometric ion-selective electrodes, general selectivity profile, electronic tongue, quantitative ion analysis

## Abstract

The present paper addresses the development and use of a new potentiometric electronic tongue for both qualitative and quantitative characterization of natural mineral waters. The electronic tongue is particularly related to the conductivity and ion content of/in the water sample. The analytical system is based on six ion-selective electrodes whose membranes are formulated to provide either cationic or anionic response and considering plasticizers with different dielectric constants (bis(2-ethylhexyl) sebacate, 2-nitrophenyl octyl ether or tricresylphosphate), while keeping the polymeric matrix, i.e., poly(vinyl chloride). Notably, the absence of any ionophore in the membrane provides a general response profile, i.e., no selectivity toward any special ion, which is convenient for the realization of an effective electronic tongue. The dynamic response of the tongue toward water samples of different chemical compositions and geographical locations has been obtained. At the optimized experimental conditions, the tongue presents acceptable repeatability and reproducibility (absence of hysteresis). The principal component analysis of the final potential values observed with the six electrodes allows for the differentiation and classification of the samples according to their conductivity, which is somehow related to the mineralization. Moreover, quantitative determination of the six main ions in the water samples (i.e., chloride, nitrate, hydrogen carbonate, sulfate, sodium, calcium, and magnesium) is possible by means of a simple linear calibration (and cross-validation) model.

## 1. Introduction

Natural mineral waters are identified by a specific mineral content and can be classified attending to elementary analysis. The provenance of a mineral water directly influences its composition, and hence, it is the ultimate responsible of any ascribable effect and/or characteristic. For example, the taste of mineral water has been demonstrated to depend on the chemical composition, and more specifically on the salt content, with both cations and anions contributing to a different extent [1,2]. Moreover, the possibility of mineral waters providing certain health benefits according to its chemical composition has been investigated by some authors [3,4,5]. Overall, it would be interesting to obtain trustable correlations between the mineral water composition, mainly in terms of ions, and the ascribable (e.g., flavor and health benefits) to facilitate a better exploitation path. A good strategy would be the provision of devices able to quantify the concentration of the main ions in a fast manner and being compatible with on-line analysis for effective implementation in control chains and other analytical setups.

Electronic tongues (ETs) are analytical tools that consist of a combination of sensors with a mathematical treatment that is developed based on measurements of a pool of samples. These samples must be carefully selected to build up the ET, being a set that is representative of any sample that would be further analyzed. Effectively, the final aim of the ET can be (i) the qualitative or quantitative differentiation of every sample, (ii) classification according to specific criterion (such as an important characteristic present or not in the sample) or (iii) combination of quantification with classification purposes. Regarding the type of sensors included in ETs, seemingly, those presenting a low selectivity profile (i.e., non-specific sensors) provide rich responses that are suitable for the development of an appropriate mathematical treatment [6]. Nevertheless, sensors that are highly selective for only one compound have been also utilized in ETs, being indeed able to quantify those specific compounds [7].

In the past years, the great utility of ETs to characterize and classify drinking water samples has been demonstrated, standing up the use of electrochemical sensors in general and potentiometric ion-selective electrodes (ISEs) in particular. Mahato et al. reported on an ET based on functionalized polymer membrane electrodes that was able to classify five branded drinking waters [8]. Atas et al. worked on an ET for the simultaneous determination of calcium, magnesium, potassium and ammonium ions in water samples, including one mineral water [7]. The organoleptic analysis of drinking water was achieved by Gutiérrez-Capitán et al. via an ET composed of six ion-selective field effect transistor sensors, a conductivity sensor and redox potential sensor together with two amperometric electrodes, a gold microelectrode for chlorine and an oxygen sensor [9].

The group of Legin, who is pioneer in the development and application of ETs, reported on the determination of conductivity, dry residual and SiO_2_ content in mineral waters [10]. The group of Del Valle developed an ET that was applied to the determination of three anions (chloride, nitrate and hydrogen carbonate) in water samples from different sources (well waters, spring waters, and tap waters) [11]. This group also reported on an ET that permitted the determination of two cations (calcium and magnesium) in mineral waters [12], and another one able to detect ammonium, potassium and sodium in some surface water samples [13]. The group of Bratov [14] tested an ET for the analysis of potassium, sodium, calcium and chloride in different brands of mineral waters. The mentioned works are representative for an overview of the entire context of water analysis based on ETs, clearly revealing the trend of classification purpose and some recent efforts toward (partial) quantitative analysis [15,16]. Nevertheless, it came to our attention the lack for effective sodium detection in mineral waters, which is mentioned in the most recent literature [12].

In this paper, we investigate the dynamic response of six potentiometric electrodes (i.e., potential redout at zero current conditions) simply based on the combination of either an anion- and a cation-exchanger together with three different plasticizers toward different mineral water samples. Various experimental conditions and protocols are researched, aiming at reproducible responses that do not show hysteresis effect. The goal is to achieve a potentiometric ET that allows for the classification of mineral waters together with the quantitative determination of the major ions. All this performed in a fast way and through the utilization of basic ISEs with a non-selective potential profile, i.e., not using expensive ionophores, and with a chemometric treatment that is relatively simple.

## 2. Materials and Methods

### 2.1. Materials and Instruments

Poly(vinyl chloride) (PVC) of high molecular weight, 2-nitrophenyl octyl ether (NPOE), bis(2-ethylhexyl) sebacate (DOS), potassium tetrakis(4-chlorophenyl)borate (KTClPB), tridodecylmethylammonium chloride (TDMACl), tetrahydrofuran (THF) and tricresylphosphate (TCP) were purchased from Sigma Aldrich (Germany). Analytical reagent grade salts sodium chloride (NaCl), sodium nitrate (NaNO_3_), sodium sulfate (Na_2_SO_4_), sodium hydrogen carbonate (NaHCO_3_), magnesium chloride (MgCl_2_) and calcium chloride (CaCl_2_) were obtained in Alpha Aesar (Germany). All solutions were prepared in 18.2 MΩ cm^−1^ doubly deionized water (Milli-Q water systems, Merck Millipore, Germany). Several brands of bottled natural mineral waters and a sample of tap water were analyzed in this work. Natural waters were selected to be originated from different provinces of Spain to cover different mineral content: Badajoz (B), Cuenca (C1 and C2), Gerona (G), Granada (Gr), Murcia (M), Segovia (S) and Teruel (T). The tap water sample was collected from the tap in our laboratory in the Campus of Espinardo, Murcia (MT).

Electrode potentials were recorded against an Orion 90–02 double junction silver-silver chloride reference electrode and using a multichannel potentiometer that was a home-made high-impedance data acquisition 16-channel box connected to a personal computer by USB. The outer compartment of the reference electrode was filled with 1.0 × 10^−1^ M KCl solution. The potential readouts (at zero current conditions) were acquired at 1 s frequency.

The conductivity electrode CM 35+ with 5060 cell (Crison, Spain) was used to detect the conductivity of each water sample. Additional ion concentration analyses were accomplished by ion chromatography (Cl^−^, SO_4_^2−^ and NO_3_^−^, ICS-2100, Dionex, Thermo Fischer, Spain), acid–base titration with hydrochloric acid (HCO_3_^−^) and inductively coupled plasma (Na^+^, Ca^2+^ and Mg^2+^, ICP Perkin Elmer Optima 200DV, Perkin Elmer Inc., Seer Green, UK).

### 2.2. Preparation of the Ion-Selective Electrodes

Six membranes of different compositions were prepared by using several plasticizers (NPOE, TCP or DOS) and ion exchangers (cation-exchanger KTClPB or anion–exchanger TDMACl). The membranes were prepared by dissolving approx. 100 mg of PVC, 200 mg of the corresponding plasticizer and 2 mg of the ion exchanger in 3 mL of THF. The exact composition of each membrane is presented in Table 1. Each solution was poured into a Fluka glass ring (inner diameter 28 mm, height 30 mm) placed on a Fluka glass plate and allowed to settle overnight until total evaporation of THF had occurred, thus obtaining the corresponding plasticized PVC membrane. A 6 mm diameter piece was cut out with a punch (Fluka, Spain) and incorporated into an ISE electrode body (Fluka) containing 1.0 × 10^−3^ M KCl as the internal filling solution. The electrodes were conditioned in KCl 1 × 10^−3^ M solution until they reached a constant potential baseline (±0.5 mV/min). Normally, overnight conditioning ensures such a response. When not in use, the electrodes were kept in KCl 1 × 10^−3^ M solution. Notably, the utilized ISEs were already reported in the literature, and the figures of merit related to calibration graphs of individual cations/anions were assessed [17,18]. Accordingly, such experiments have not been herein performed, and so, the electrodes were directly utilized to measure in the water samples.

### 2.3. Procedure for the Analysis of the Samples

First, the conductivity of each water sample was analyzed, and the observed values are collected in Table 2. A wide range in conductivity values was found, with the least conductive being the B water and the most conductive being the tap water (MT). In principle, a larger conductivity is associated with a larger ion content of the water sample. Nevertheless, each sample was analyzed by elemental analysis to discern the ions that are responsible for such differences in conductivity. The results are shown in Table 3. As observed, the tap water (MT) presents the higher content for all the ions except for nitrate, which is similar in all the water samples. A comparison between the composition of all the natural mineral waters shows that the M sample is characterized by a high content for all the ions, except for nitrate. C1 and C2 water samples possess high content in calcium and hydrogen carbonate. G and Gr are quite similar in composition except for a larger content of chloride in sample G. B is the sample with a lower ion content, together with S and T, which agrees with the conductivity measurements. Notably, there are two samples with a rather high sodium, chloride, and sulfate content: the tap water MT and the mineral water M, both samples arising from the same province.

**Figure 1:**
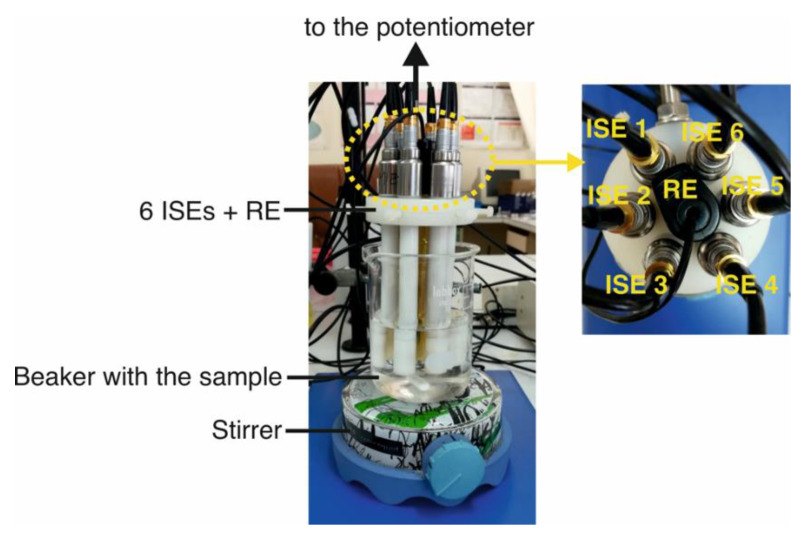
Illustration of the experimental setup.

The six-electrode array was immersed in 50.0 mL of 1.0 × 10^−6^ M KCl solution with constant stirring (300 rpm) for 50 s (see Figure 1). The potential recorded by each electrode is visualized in the software. Then, a 10.0 mL aliquot of the water sample to be analyzed was added. The potential of each of the six electrodes was registered during the entire process, and the potential values finally reached after 100 s of the beginning of the experiment were considered for following data processing. Before measuring the next sample, the electrodes were gently washed with water and dried. Each natural water sample was analyzed in triplicate, while a total number of nine measurements were collected for the tap water sample (triplicate signals at the beginning, within and at the end of the entire analysis). The generated pool of signals allowed for reproducibility/repeatability and hysteresis evaluation, while providing a reasonable analysis time for all the samples’ measurements and thus, the generation of the data needed for the ET. Then, the potential signals were processed using the standard data analysis tools (numpy, scikit-learn, pandas, matplotlib) of the Python language, for principal component analysis and linear regression.

## 3. Results

### 3.1. Investigation of the Response of the Electrodes Conforming the ET toward an Accurate Measurement Protocol

The six-electrode array was constructed based on two types of electrodes: three ISEs containing a cation exchanger in the membrane (1, 3 and 5 in Table 1) and other three ISEs with anion exchanger instead (2, 4 and 6 in Table 1), providing hence cationic and anionic responses, respectively [17]. Then, for each class of electrode, plasticizers with different dielectric constants were used (NPOE, TCP and DOS with ε = 24, 7 and 4, respectively) [18]. None of the six ISEs contained a selective receptor or ionophore, and therefore, the expected selectivity profiles are expected to be based on regular partition fundamentals at the sample-membrane interface, which is essentially dictated by the ion lipophilicity (i.e., the more lipophilic the more tendency to enter the membrane). The selection of electrodes with a low selectivity profile is common in the development of ETs, as described in the Introduction section. Furthermore, the presence of a different plasticizer in each membrane aims at generating slight differences in the overall response of each ISE toward a series of cations or anions. Accordingly, the overall response of each ISE will be in principle different when analyzing distinct water samples, being hence suitable to build up an effective ET.

Several experimental procedures were tested to achieve reproducible time traces of the potential response of each of the six ISEs in the different mineral water samples. The best results were obtained by immersing the electrode array in 50.0 mL of 1.0 × 10^−6^ M KCl concentration for 50 s, followed by the addition of 10.0 mL of the corresponding mineral water sample and recording the response over another 50 s (i.e., total analysis time of 100 seconds per sample). Otherwise, the response provided by the ISEs was found to display a certain hysteresis effect and the baseline changed after testing each sample, which would strongly affect the results provided by the ET in the way of inappropriate precision.

**Figure 2:**
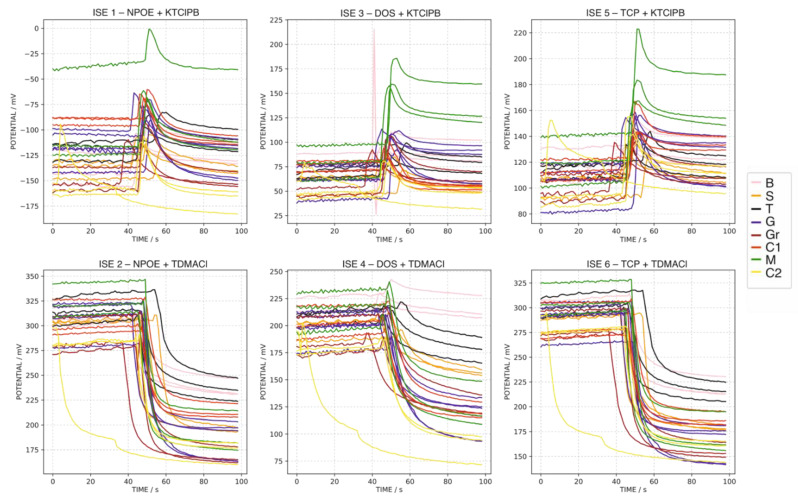
Triplicate dynamic potential responses of ISEs 1–6 observed for all the water samples, except MT, applying the first measurement protocol (MilliQ water background).

**Figure 3:**
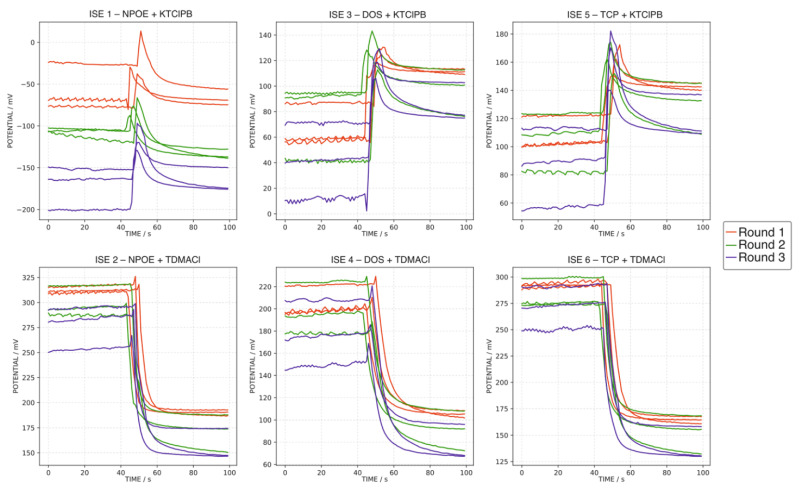
Triplicate dynamic potential responses of ISEs 1–6 observed for the MT sample at the beginning, middle and end of the entire experimental testing of the sample pool, applying the first measurement protocol (MilliQ water background).

In a first approach, the water samples were analyzed as follows. The electrode array was immersed in 50.0 mL of MilliQ water for 50 s followed by the addition of 10.0 mL of the corresponding water sample and recording the response over another 50 s. The results are presented in Figure 2. In addition, to evaluate any hysteresis issue, the same sample (i.e., the tap water sample, MT) was measured in triplicate at the beginning, in the middle and at the end of the analysis to investigate any possible hysteresis effect. Figure 3 shows the responses observed for the MT at the three different moments over the entire analysis. As observed in both figures, the absence of an appropriate reproducibility of consecutive measurements was evident, with the additional presence of a certain hysteresis effect when measuring the same sample over the entire analysis experiment. In essence, the membranes incorporated in the ISEs initially contained either the potassium or chloride salt of the corresponding ion exchanger, which are exchanged at different degrees during the exposition of the membrane to each sample, and hence, the initial state of the membrane (and thus the corresponding baseline) changes after each water sample is analyzed.

Accordingly, we modified the experimental protocol to preserve the initial state of each membrane as much as possible within the measurements. Otherwise, the overall precision of the ET would be rather questionable. A good performance was achieved using 1.0 × 10^−6^ M KCl solution rather than MilliQ water to obtain the initial baseline. It is expected that, during the first 50 s with the electrode immersed in this solution, the presence of potassium and chloride ions in the solution regenerates the initial state of the membrane through a reverse exchange to that experienced in the previously tested sample. Different KCl solutions (in the range from 1.0 × 10^−6^ M to 1.0 × 10^−3^ M KCl) were tested to achieve this purpose. Notably, in general terms, the potential change observed after adding the water sample was lower when increasing the KCl concentration in the background solution due to the competition between the potassium or chloride ion in the background with those ions in the water sample, i.e., more marked interference as the KCl concentration was increased. As a result, 1.0 × 10^−6^ M KCl solution was selected for further studies.

**Figure 4:**
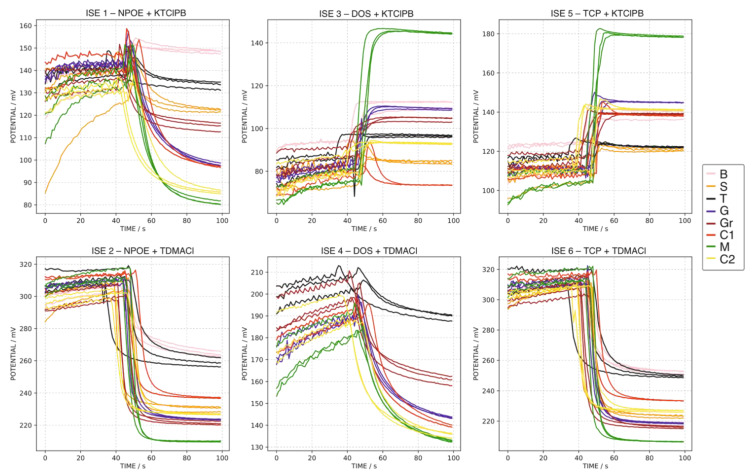
Triplicate dynamic potential response of ISEs 1–6 observed for all the water samples, except MT, applying the second measurement protocol (1.0 × 10^−6^ M KCl background).

**Figure 5:**
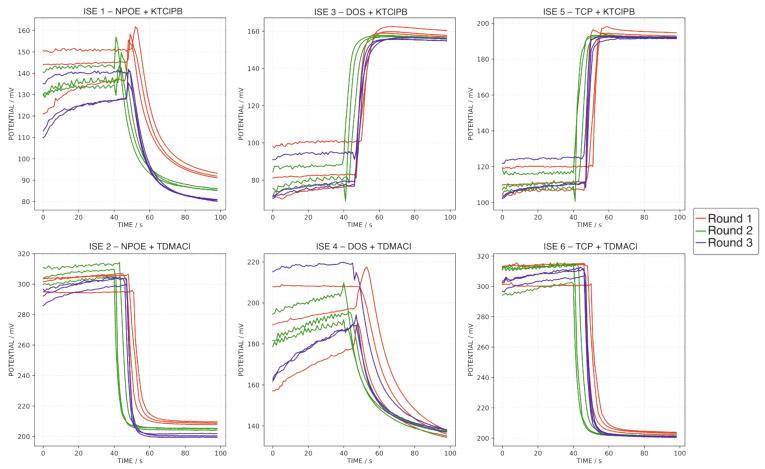
Triplicate dynamic potential responses of ISEs 1–6 observed for the MT sample at the beginning, middle and end of the entire experimental testing of the sample pool, applying the first measurement protocol (1.0 × 10^−6^ M KCl background).

Following the just-described procedure, every water sample was analyzed in triplicate (Figure 4), including the MT at the beginning, middle and end of the test (Figure 5). As observed, despite slight changes appearing in the baseline and the total potential change, the final potential was fairly maintained between repetitive measurements for all the electrodes (standard deviations of 0.81, 0.85, 0.48, 0.48, 0.50 and 1.99 mV for ISEs 1–6, respectively). In addition, the good reproducibility of the final potential of the MT sample was remarkable (0.51, 0.77, 0.54, 0.40, 0.70 and 0.99 mV for ISEs 1–6, respectively), pointing out the absence of hysteresis. Therefore, the last potentials acquired for each sample (at 100 s) were used for the further characterization of the water samples’ pool, because of the acceptable reproducibility and demonstrated absence of the hysteresis effect.

Inspecting now the overall potential signals provided by each ISE, as a general trend, the more ion content in the sample, the higher the potential jump that was observed. Moreover, according to the ion concentrations observed with the IC, titration and ICP measurements, it is evident that the main differences in the signals mainly come from the distinct Ca^2+^ and HCO_3_^−^/SO_4_^2−^ concentrations in the samples (see Table 3). Another interesting aspect to be discussed is the monotonicity nature of the observed potentiometric signals. For the ISEs presenting anionic response (2, 4 and 6), the potential decreased in a continuous way until reaching a (close to) steady-state value. In other words, these ISEs showed a monotonic response. Furthermore, the response time was relatively fast for the ISEs prepared with TCP and NPOE as plasticizer compared to the DOS. In addition, the responses for the TCP and NPOE ISEs were rather similar between them for all the tested samples.

Regarding the ISEs displaying a cationic response (1, 3 and 5), most of the signals presented a remarkable non-monotonic behavior characterized by an initial period in which the signal increased followed by a decreasing trend until a (close to) steady-state value. This kind of non-monotonic signal has been previously reported for ISEs prepared without any ionophore, being analogous to those used in the present work [19,20]. The response of the ISE 1 based on NPOE as the membrane plasticized is of particular interest because the final potential for most of the samples is remarkably lower than that observed for the baseline in the 1.0 × 10^−6^ M KCl background solution. A similar behavior was observed for the responses collected by the first measurement protocol (Figure 2) but obtaining a final potential rather similar to or a slightly lower than that recorded in MilliQ water background.

Notably, the response of a similar electrode has been deeply studied in a recent work by our group [19]. In essence, when several cations were tested individually at increasing concentrations, non-monotonic signals were observed for some of them, namely Na^+^, Ca^2+^ and Mg^2+^. The seminal paper also mentions that other authors have reported on similar observations, and moreover, theoretical models have been established to explain such a behavior. Additionally, in the present work, the signals presented overall potential changes that involve a final potential value lower than the initial one.

**Figure 6:**
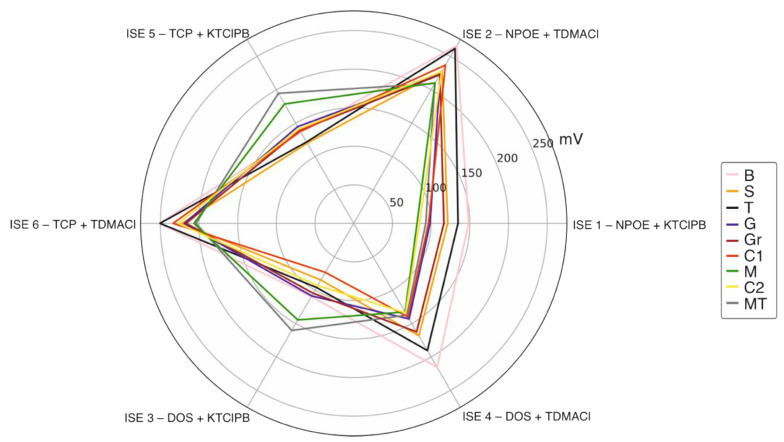
Radar plot of the final potential displayed by each electrode (1–6) in each water sample. Each line connects the data related to the same sample.

Figure 6 depicts the radar plot of the final potential displayed by each electrode (1–6) in each water sample (the first measurement of the replicate analysis), being those data related to the same sample connected with a line. As observed, the lines were found to cross between them, which pointed out that the electrodes presented cross-selectivity behavior. This aspect is more noticeable for the cation-selective electrodes (2 and 4) than for the anion-selective electrodes (1, 3 and 5).

A systematic correlation study of the final potentials obtained with the six electrodes for all the water samples showed that some of the observed responses were not independent between them (Table 4). Considering a threshold value of 0.80 [21], a significant, positive correlation between the potentials provided by the NPOE-TDMACl and DOS-TDMACl ISEs (electrodes 2 and 6, respectively), and between the NPOE-TDMACl and TCP-TDMACl ISEs (electrodes 1 and 3. respectively) was evidenced when the Pearson correlation coefficients were evaluated (0.84 and 0.99, respectively). Moreover, and considering that the values are calculated for a specific pool of samples, using either NPOE or TCP as plasticizers in anion responsive ISEs is enough to gather the same information. Notably, this conclusion should be carefully considered regarding whether the ET would like to be used for a different set of water samples, because the mentioned correlations will strongly depend on the sample set. For the cation responsive ISEs, the TCP-KTClPB and DOS- KTClPB ISEs (electrodes 3 and 5, respectively) also presented a positive correlation, but close to the threshold limit. Moreover, some cross-correlations between ISEs that response to either anions or cations were evident, while not expected: NPOE-KTClPB with NPOE-TDMACl (ISE 4, coefficient = 0.83) and with DOS-TDMACl (ISE 6, coefficient = 0.97).

### 3.2. Principal Component Analysis

**Figure 7:**
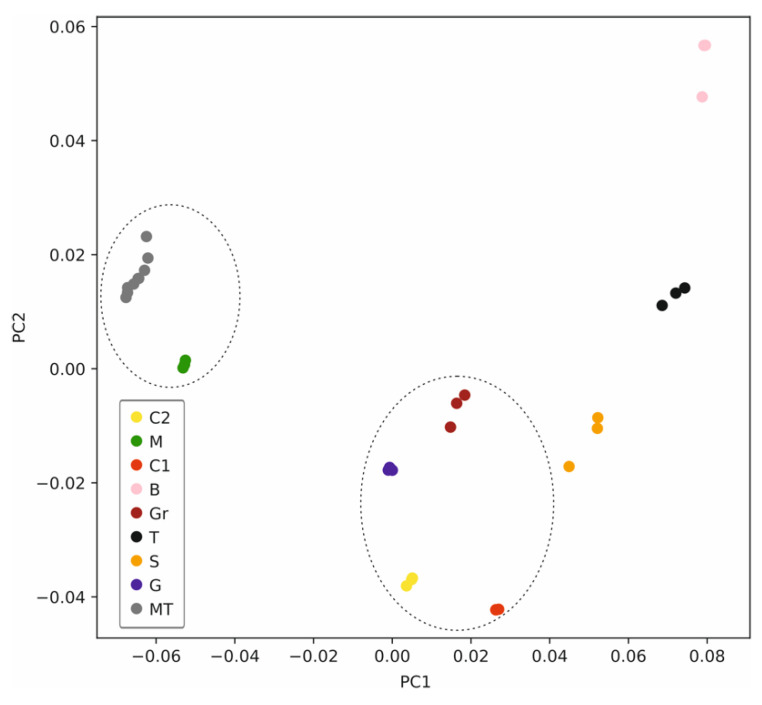
Principal component analysis. Plot of PC1 versus PC2 calculated for the pool of samples and including the three MT repetitions along the analysis.

Figure 7 presents the principal component analysis (PCA) of the signal pool observed for all the tested water samples (in triplicate) together with the values for the MT sample measured along the entire analysis, i.e., the signals in Figure 4 and Figure 5. Advantageously, the principal components 1 and 2 (PC1 and PC2) were found to represent 96.5% of the total variance, and thus, these two PCs together will quite accurately define the differences and similarities between the analyzed samples. In addition, the points corresponding to each sample are well separated between them, which allows for an easy differentiation. More specifically, the percentages of captured variances for each PC were 77.1%, 19.3%, 2.6%, 0.5% and 0.3% for PC1–PC5, whereas the percentages of accumulated variance were 77.1%, 96.5%, 99.1%, 99.6% and 100.0% for PC1–PC5. Remarkably, the six points corresponding to the MT sample measured along the entire analysis are close between them, indicating an acceptable reproducibility of the measurements and negligible hysteresis influence.

**Figure 8:**
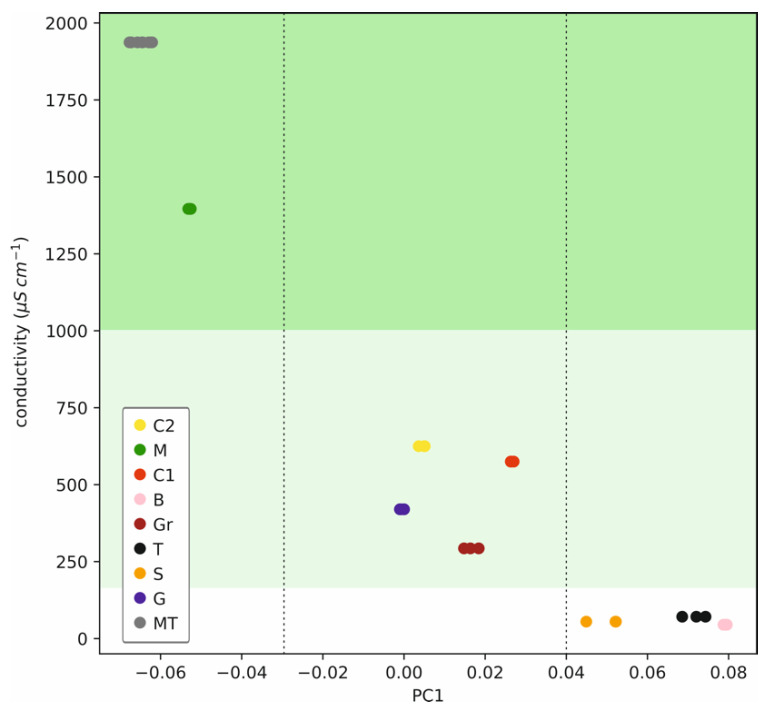
Plot of the conductivity of each sample versus PC1 for the pool of samples and including the three MT repetitions along the analysis. Regions are distinguished for high (dark green), medium (light green) and low sample conductivity.

We hypothesized that the samples were grouped into three categories in Figure 7 (group 1: MT and M; group 2: C2, C1, Gr, and G; group 3: B, S and T, according to the drawn ellipses). Then, we deeply explored possible relationships of either PC1 or PC2 with the sample conductivity, attempting to understand the potential of the PCA for the further quantification of ion content in the sample and hence to verify our classification hypothesis. Effectively, the value for PC1 was found to be related to the conductivity of the water sample, and hence, three different regions (for high, medium and low conductivity) were distinguished in the plot of conductivity versus PC1 (Figure 8).

More in detail, those samples with the lower conductivity values (<200 µS cm^−1^: B, S and T) presented a PC1 ranging from 0.04 to 0.09, whereas the samples with higher conductivity (from 1000 to 2000 µS cm^−1^: MT and M) displayed PC1 values ranging from −0.07 to −0.04. Samples with intermediate conductivity appear in the middle part of Figure 8, with PC1 values ranging from 0 to 0.03. Of note is that the S sample is separated from B and T, which can be due to its higher content in nitrate (Table 3). It is remarkable how the ET can differentiate between the three samples with the lower conductivity and labelled by the corresponding manufacturer as “low mineralization water”. It would be interesting to further utilize the ISE system here developed to analyze more water samples with low conductivity (and thus mineralization) to understand whether the ET can be particularly used to classify these samples according to their origins (e.g., geography, type of stone in the aquatic resource, etc.).

Next, the potential of the ET to predict the quantitative analysis of the ion concentration in the water samples was investigated. The elemental ion analysis displayed in Table 3 was used to construct a simple mathematical model based on linear regression. The electrode calibration was performed using the entire pool of samples, and the quality of the concentration predictors was evaluated by cross-validation. The concentration predictor is expressed according to Equation (1):

(1) $$c_{n}=\sum_{k=1}^{n}w_{k}E_{k}+\mathrm{offset}$$

where $c_{n}$ is the concentration of each ion *(n)* expressed in mg/L, $w_{k}$ stands for the coefficients obtained by linear fitting providing the minimum quadratic error, $E_{k}$ (expressed in mV) is the final potential value of each electrode *(k)*, and offset is the ion concentration corresponding to a zero potential readout.

**Figure 9:**
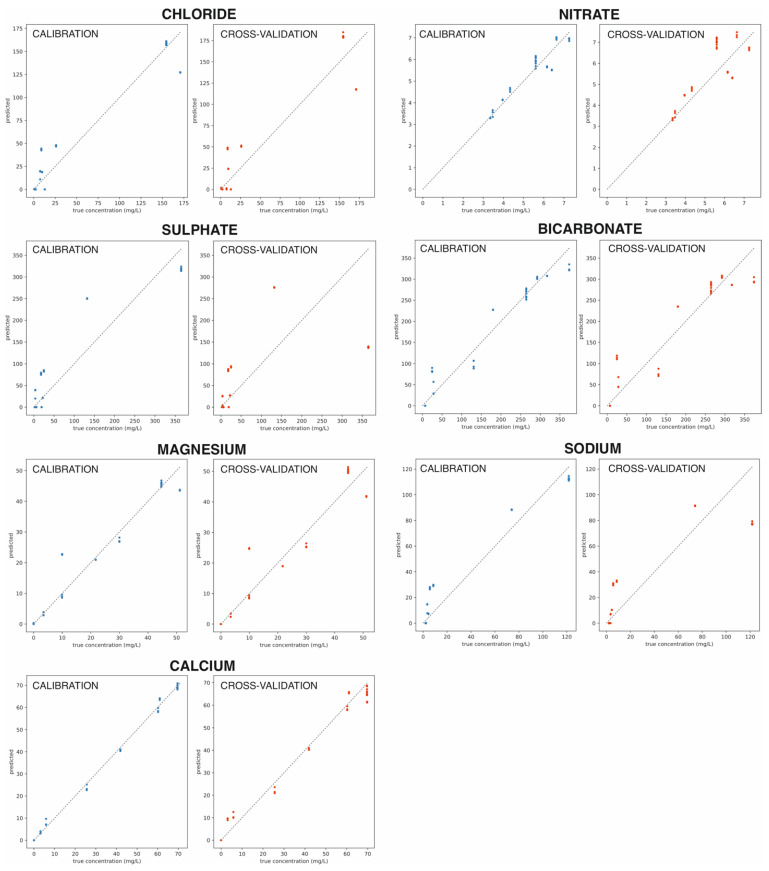
Calibration and cross-validation models (predicted versus true concentration values) obtained for all the ions tested in the mineral water samples.

Table 5 presents the best fit weights ($w_{k})$ for the predictor of each ion concentration, calculated using a dimension reduction. In all cases, PCR (principal component regression) and PLS (partial least squares) considering from one to four components were investigated. Table 6 collects the quality parameters obtained for the best predictors for each ion that was found by using the cross-validation. Notably, the specific method selected to reduce the data dimension for each ion (PCR or PLS) is provided in the last column, with the number indicating the number of components that were used. Due to the high repeatability found for the signals (see above), the cross-validation was conducted attending to the type of water, i.e., all samples of each type of water are predicted with a model that is calibrated with all the samples of the other types. The results are shown in Figure 9. The calibration curves of the best concentration models are displayed in separate rows for each ion. The graphs at the left show the raw calibration curves, where all samples are used to build the model. Then, the cross-validation results are presented in the curves at the right, where each water sample is evaluated with a model that does not use the same type of water. Good cross-validation accuracy usually indicates that the model is not overtrained and will generalize well.

Overall, the $w_{k}$ values in Table 5 allow for simple formulas to predict the concentration of seven ions (Cl^−^, NO_3_^−^, SO_4_^2−^, HCO_3_^−^, Mg^2+^, Na^+^ and Ca^2+^) in natural mineral water samples. Nevertheless, the concentration of each ion can be predicted with different accuracy, as revealed by the R^2^ prediction scores presented in Table 6. For example, Cl^−^ and Ca^2+^ can be estimated with high accuracy in the water samples in the range of concentrations herein studied, whereas sulphate is the poorest predicted. Remarkably, this situation may change depending on the pool of samples used to develop the quantitative ET. Furthermore, the good calibration results obtained by simple linear models indicated that the developed ET provides rich information about ion concentration and that the use of more complex data processing and machine learning techniques is not needed.

## 4. Conclusions

We have presented a potentiometric ET that is able to differentiate between distinct samples of natural mineral water, even with low conductivity, i.e., mineralization. Furthermore, the potentiometric ET allows for the quantitative determination of the main ions present in the sample, namely Cl^−^, NO_3_^−^, SO_4_^2−^, HCO_3_^−^, Mg^2+^, Na^+^ and Ca^2+^, with different accuracy levels. Qualitative differentiation is achieved with the use of only two components in a PCA approach, with PC1 being related to conductivity. Quantitative analyses are achieved with a reduction of the dimension of the data matrix via either the PCR or PLS method with a different number of components (optimized for each ion). Then, cross-validation analysis was used to evaluate the quality of the quantitative prediction via a simple linear regression. The good results obtained in this work are based on the optimization of the potentiometric response of a six-electrode array toward an ample set of water samples: the response is reproducible and is without hysteresis effects.

## Figures and Tables

**Table 1:** Detailed membrane compositions and ion-selective electrodes (ISEs) labels.

Membrane	Components								ISE
	PVC		Plasticizer			Ion Exchanger			
	mg	wt.%	Compound	mg	wt.%	Compound	mg	wt.%	
1	0.1004	33.14	NPOE	0.2011	66.37	KTClPB	0.0015	0.49	1
2	0.0999	32.97	NPOE	0.1989	66.23	TDMACl	0.0015	0.50	2
3	0.1004	31.40	TCP	0.2177	68.10	KTClPB	0.0016	0.50	3
4	0.1000	32.54	TCP	0.2057	66.94	TDMACl	0.0016	0.52	4
5	0.1011	32.82	DOS	0.2047	66.46	KTClPB	0.0022	0.71	5
6	0.0730	33.06	DOS	0.1465	66.35	TDMACl	0.0013	0.59	6

**Table 2:** Conductivity of each water sample.

	MT	C1	G	M	S	T	Gr	C2	B
Conductivity (µS cm^−1^)	1937	575	420	1396	55	71	293	625	45

**Table 3:** Ion concentration obtained by IC (Cl^−^, SO_4_^2−^ and NO_3_^−^), acid–base titration (HCO_3_^−^) and ICP (Na^+^, Ca^2+^ and Mg^2+^) in the water samples.

Ion Concentration (mg L^−1^)	MT	C1	G	M	S	T	Gr	C2	B
Ca^2+^	69.6	69.6	41.9	61.0	5.8	3.1	25.6	60.2	1.0
Na^+^	121.7	2.0	8.5	74.0	2.2	2.3	5.6	4.4	3.4
Mg^2+^	44.7	21.7	10.0	51.1	1.0	3.4	9.9	30.0	1.0
Cl^−^	154.0	2.2	26.0	170.0	0.7	12.8	8.7	9.7	7.4
NO_3_^−^	5.6	4.0	6.2	6.4	6.6	3.5	7.3	4.3	3.3
SO_4_^2−^	364.9	19.2	24.8	131.0	2.1	6.3	17.9	22.3	3.7
HCO_3_^−^	264.4	317.3	179.8	292.2	28.2	24.7	130.4	373.7	7.0

**Table 4:** Pearson coefficients for correlation between each pair of electrodes.

	(#1)	(#3)	(#5)	(#2)	(#4)	(#6)
**NPOE–KTClPB (#1)**	1.00	−0.64	−0.20	0.83	0.76	0.97
**TCP–KTClPB (#3)**	−0.64	1.00	0.81	−0.59	−0.59	−0.51
**DOS–KTClPB (#5)**	−0.20	0.81	1.00	−0.33	−0.40	−0.07
**NPOE–TDMACl (#2)**	0.83	−0.59	−0.33	1.00	0.99	0.84
**TCP–TDMACl (#4)**	0.76	−0.59	−0.40	0.99	1.00	0.76
**DOS–TDMACl (#6)**	0.97	−0.51	−0.07	0.84	0.76	1.00

**Table 5:** Fitting coefficients ($w_{k})\;$ for each sensor and offset obtained for the linear model predictor for each ion.

ISE	Cl^−^	NO_3_^−^	SO_4_^2−^	HCO_3_^−^	Mg^2+^	Na^+^	Ca^2+^	K^+^
**NPOE–KTClPB (#1)**	34.3	66.4	89.7	−4112.6	−436.8	33.9	−329.9	1.5
**NPOE–TDMACl (#2)**	−436.3	−21.9	−929.7	1459.5	82.1	−329.5	4.8	−16.3
**TCP–KTClPB (#3)**	943.3	9.3	1939.9	1174.9	256.6	685.4	1432.7	33.4
**TCP–TDMACl (#4)**	−657.9	−43.3	−1404.2	2793.7	187.0	−498.3	249.3	−24.6
**DOS–KTClPB (#5)**	2061.4	26.6	2212.5	−421.1	118.7	783.8	−983.5	38.2
**DOS–TDMACl (#6)**	215.8	48.8	466.9	−2928.4	−277.3	167.4	−534.5	8.0
**Offset**	34.0	36.1	8.1	−171.0	−64.7	−0.7	−99.5	−0.2

**Table 6:** Quality parameters of the best predictors for each ion. R^2^: coefficient of determination, proportion of variance explained by the predictor. CC: correlation coefficient between true and predicted values.

	Calibration		Cross-Validation		Method
Ion	R^2^	CC	R^2^	CC	
Cl^−^	0.93	0.97	0.87	0.94	PCR 2
NO_3_^−^	0.90	0.95	0.50	0.81	PCR 3
SO_4_^2−^	0.89	0.95	0.29	0.59	PCR 2
HCO_3_^−^	0.94	0.97	0.86	0.93	PCR 3
Mg^2+^	0.94	0.97	0.89	0.95	PCR 3
Na^+^	0.95	0.98	0.75	0.90	PCR 2
Ca^2+^	1.00	1.00	0.98	0.99	PLS 4
